# Supplementary figures and images for: hdac4 mediates perichondral ossification and pharyngeal skeleton development in the zebrafish
Source: PeerJ. 2019 Jan 8;7:e6167. doi: 10.7717/peerj.6167 (PMC6329341; doi:10.7717/peerj.6167)

Page: 1 / 4  
1/10/2017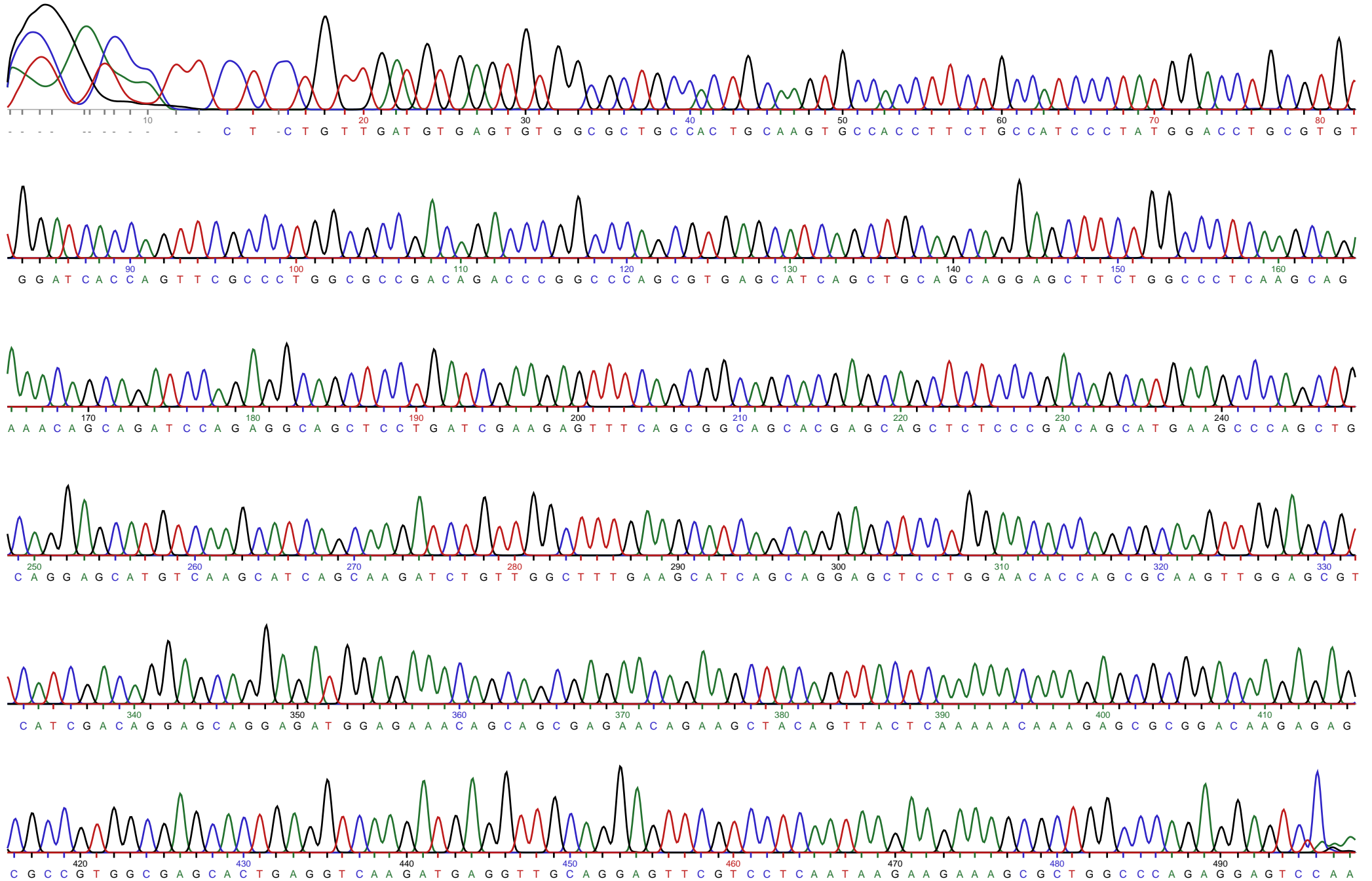

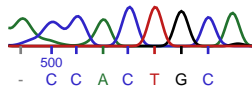

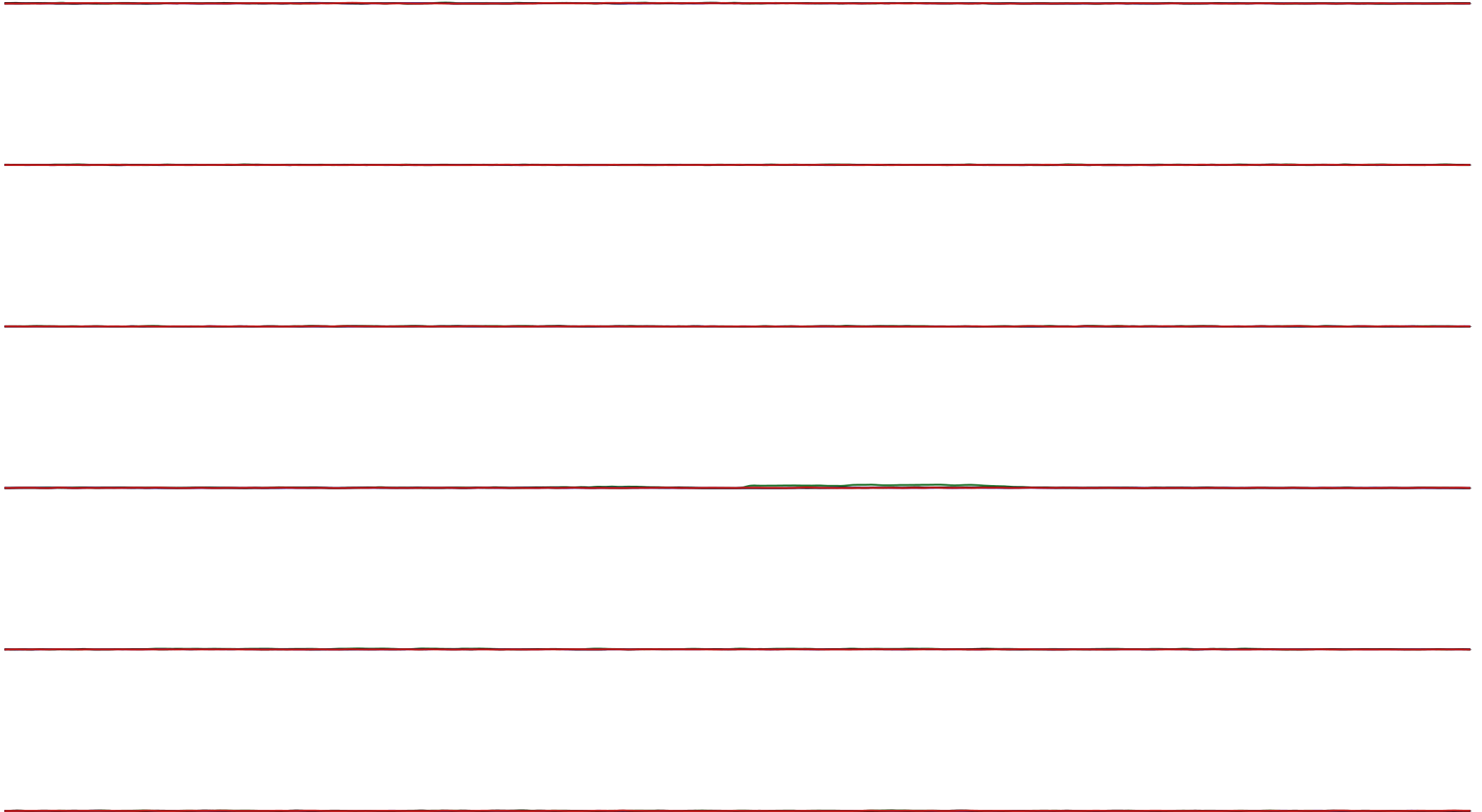

---

---

Supplement: Data S1 — Sequencing files of wild-type and mutant sibling cDNA sequenced from adult fin amputations. [file peerj-07-6167-s001.zip › WTF_PREMIX_T30323_1.pdf]

Samples: 25157  
Bases: 507  
Average spacing: 50

Page: 1 / 5  
11/10/2017

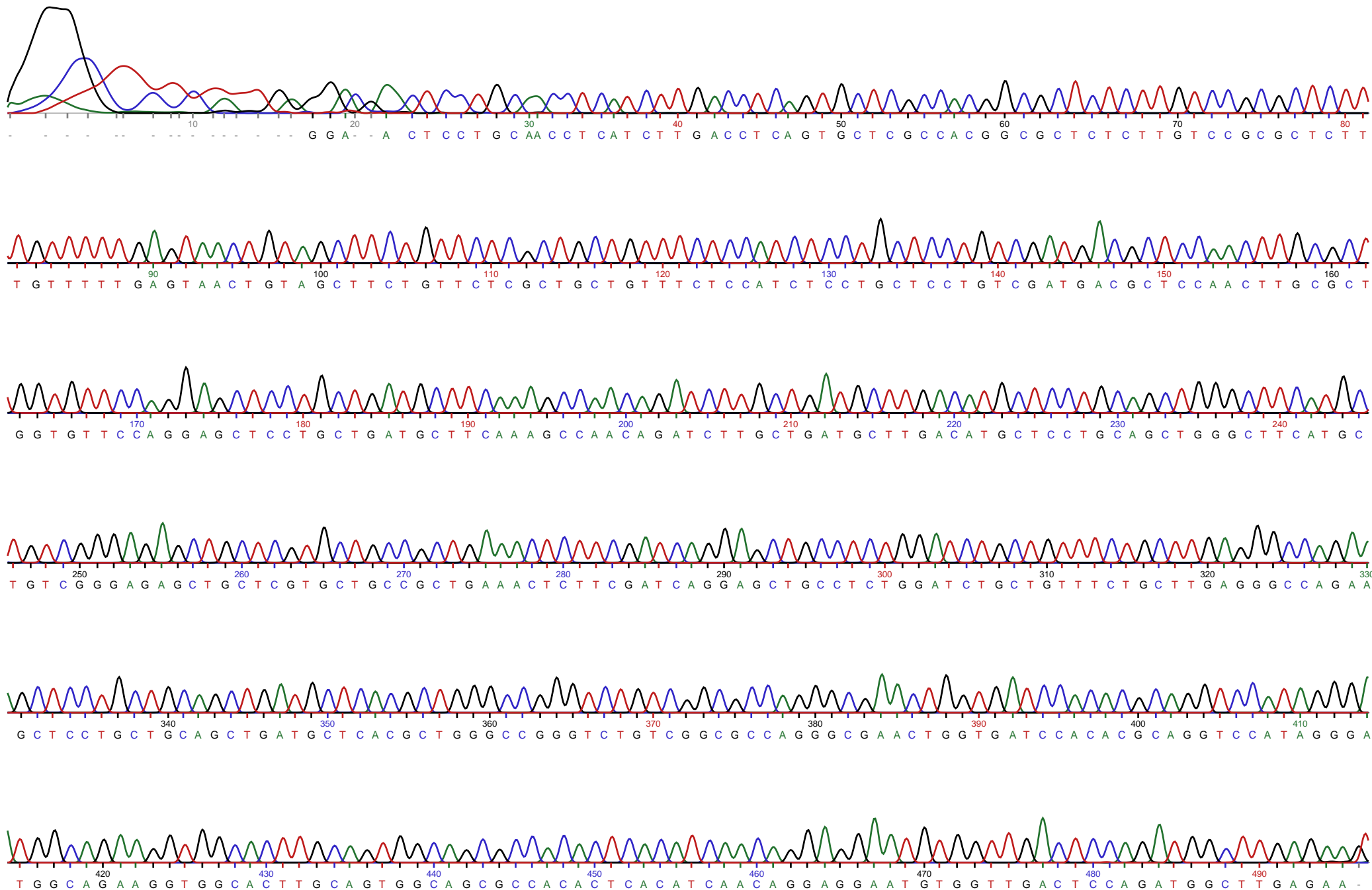

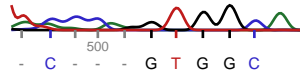

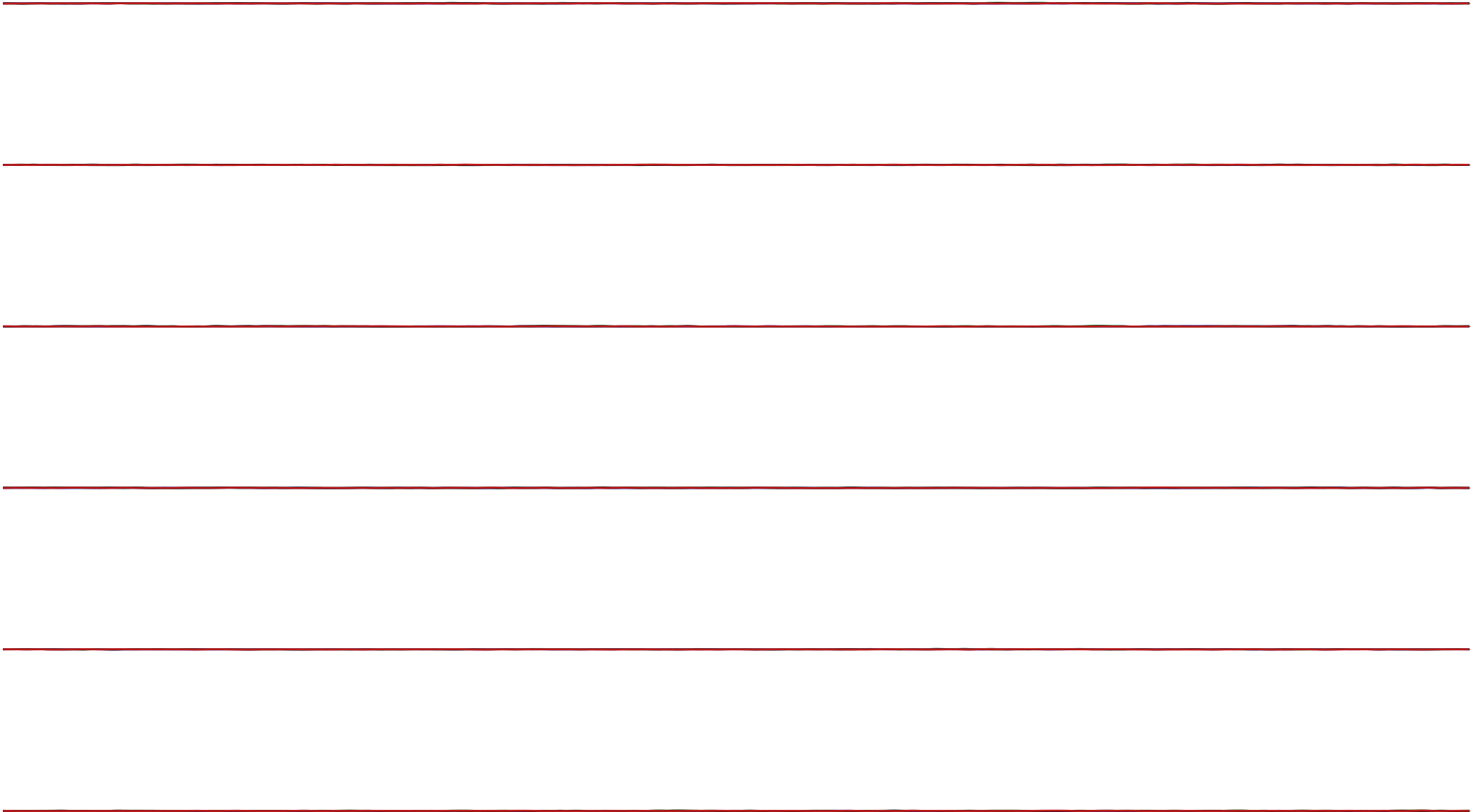

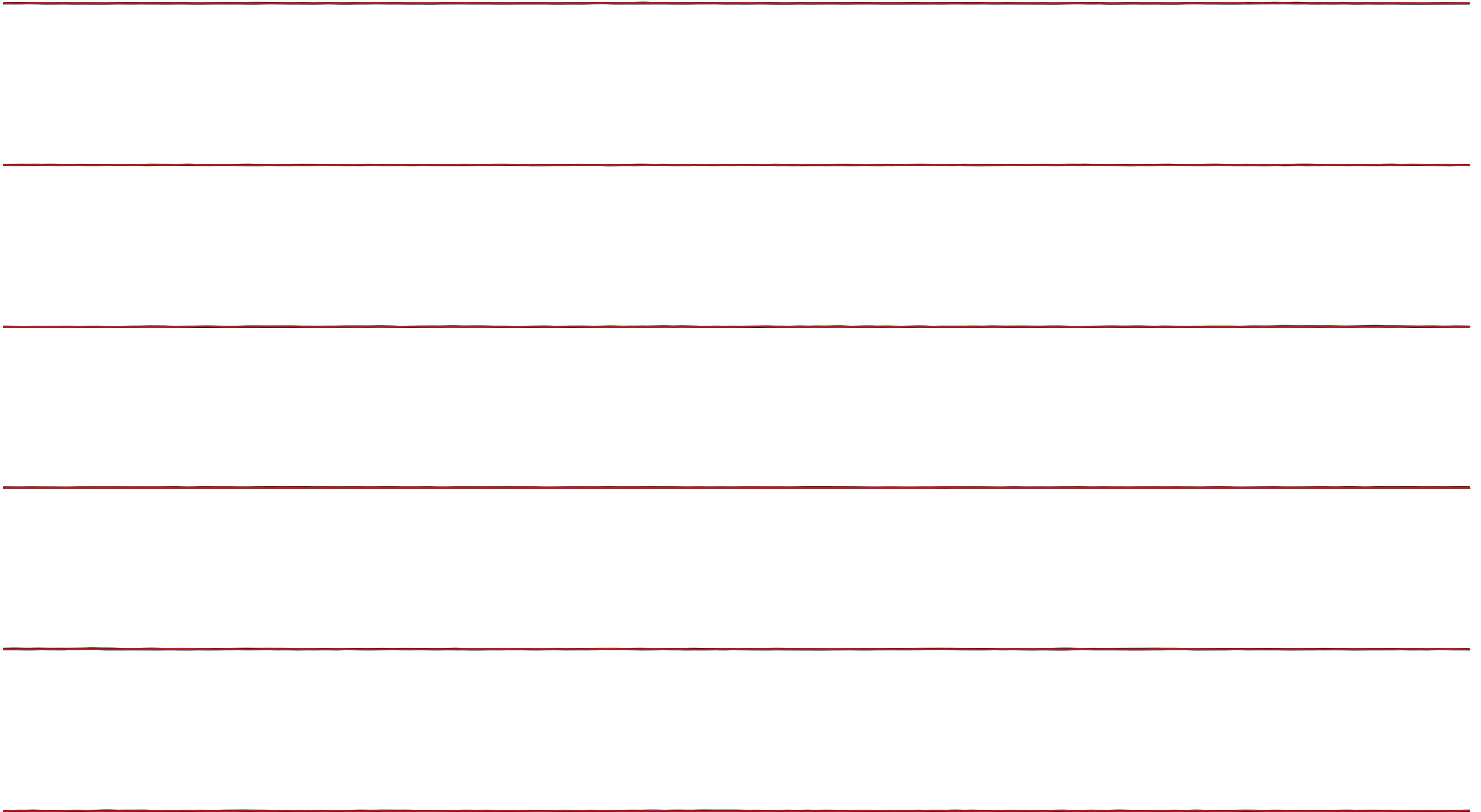

---

---

Supplement: Data S1 — Sequencing files of wild-type and mutant sibling cDNA sequenced from adult fin amputations. [file peerj-07-6167-s001.zip › WTR_PREMIX_T30324_2.pdf]

Page: 1 / 4  
1/10/2017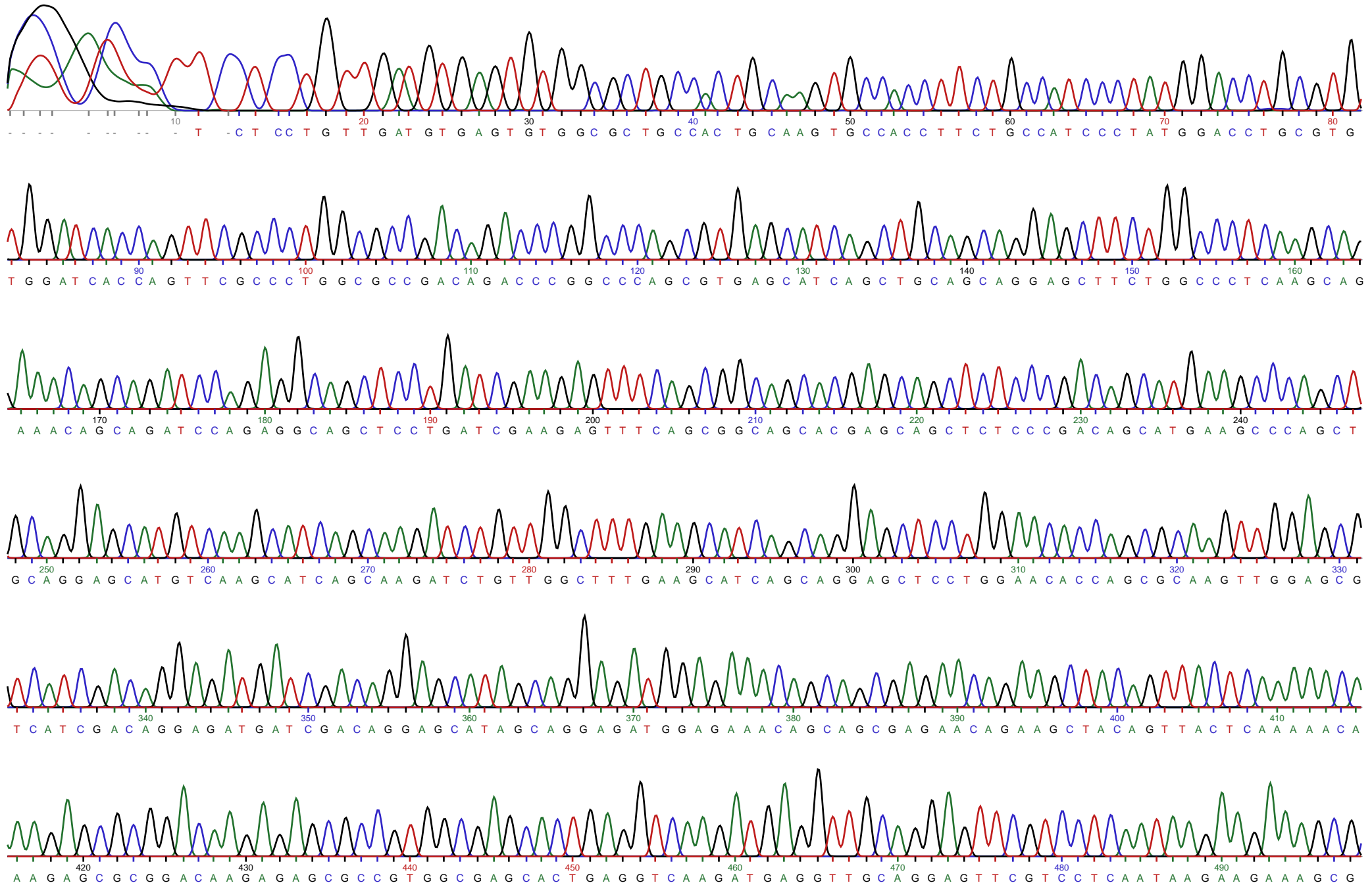

Samples: 18212  
Bases: 527  
Average spacing: 35

Page: 2 / 4  
11/10/2017

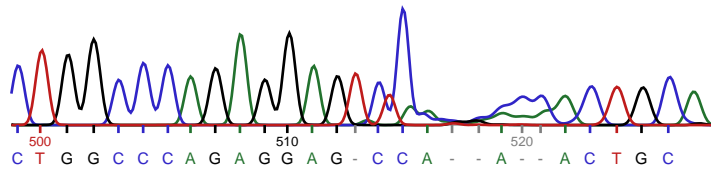

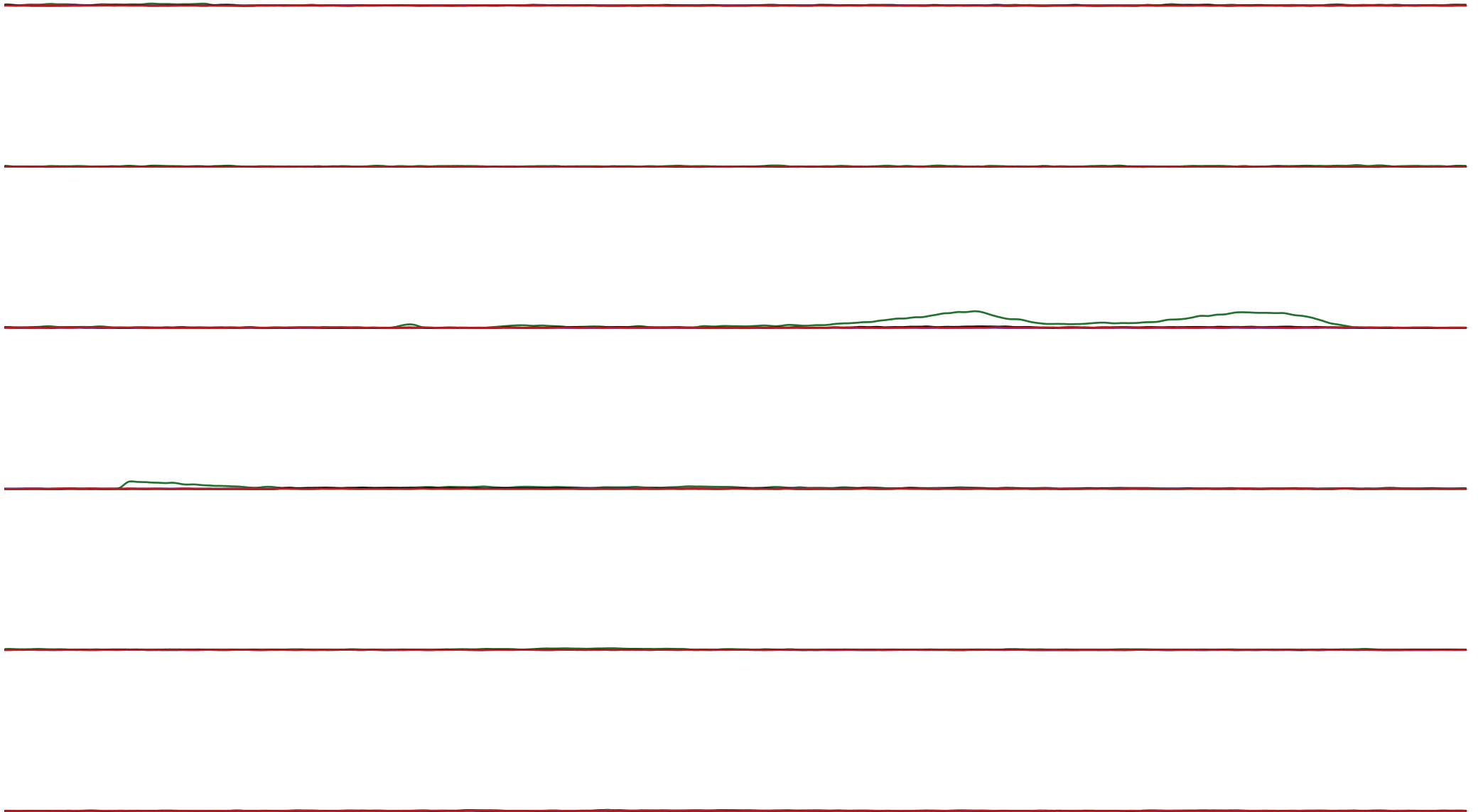

Samples: 18212  
Bases: 527  
Average spacing: 35

Page: 4 / 4  
11/10/2017

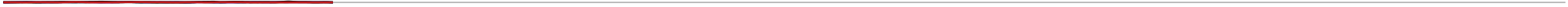

Supplement: Data S1 — Sequencing files of wild-type and mutant sibling cDNA sequenced from adult fin amputations. [file peerj-07-6167-s001.zip › mutF_PREMIX_T30325_3.pdf]

Samples: 12969  
Bases: 1056  
Average spacing: 13

Page: 1 / 3  
11/10/2017

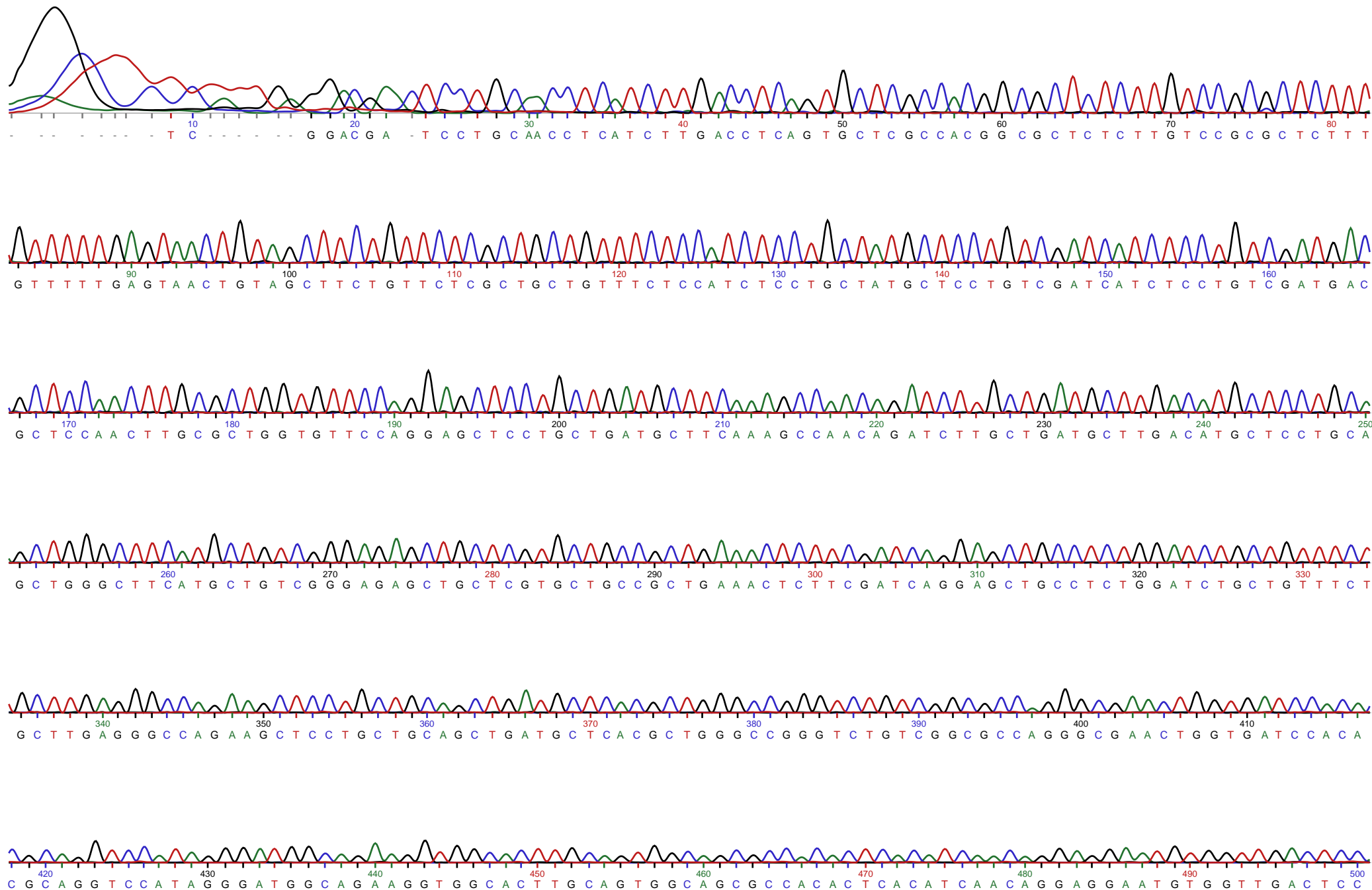

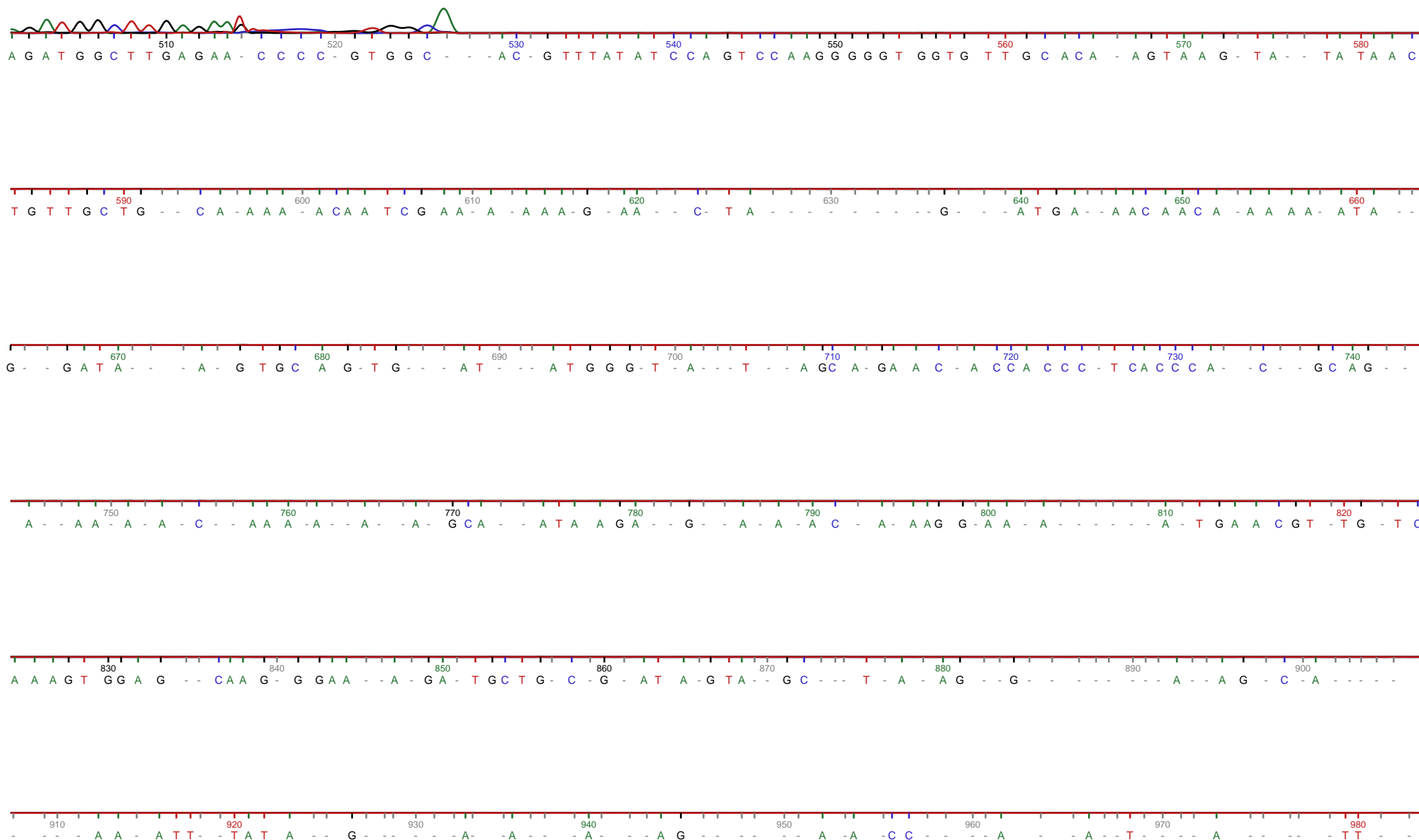

|                  |       |
|------------------|-------|
| Samples:         | 12969 |
| Bases:           | 1056  |
| Average spacing: | 13    |

Page: 3 / 3  
11/10/2017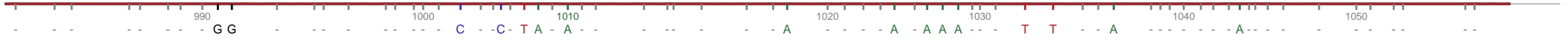

Supplement: Data S1 — Sequencing files of wild-type and mutant sibling cDNA sequenced from adult fin amputations. [file peerj-07-6167-s001.zip › mutR_PREMIX_T30326_4.pdf]

Page: 1 / 4  
6/30/2018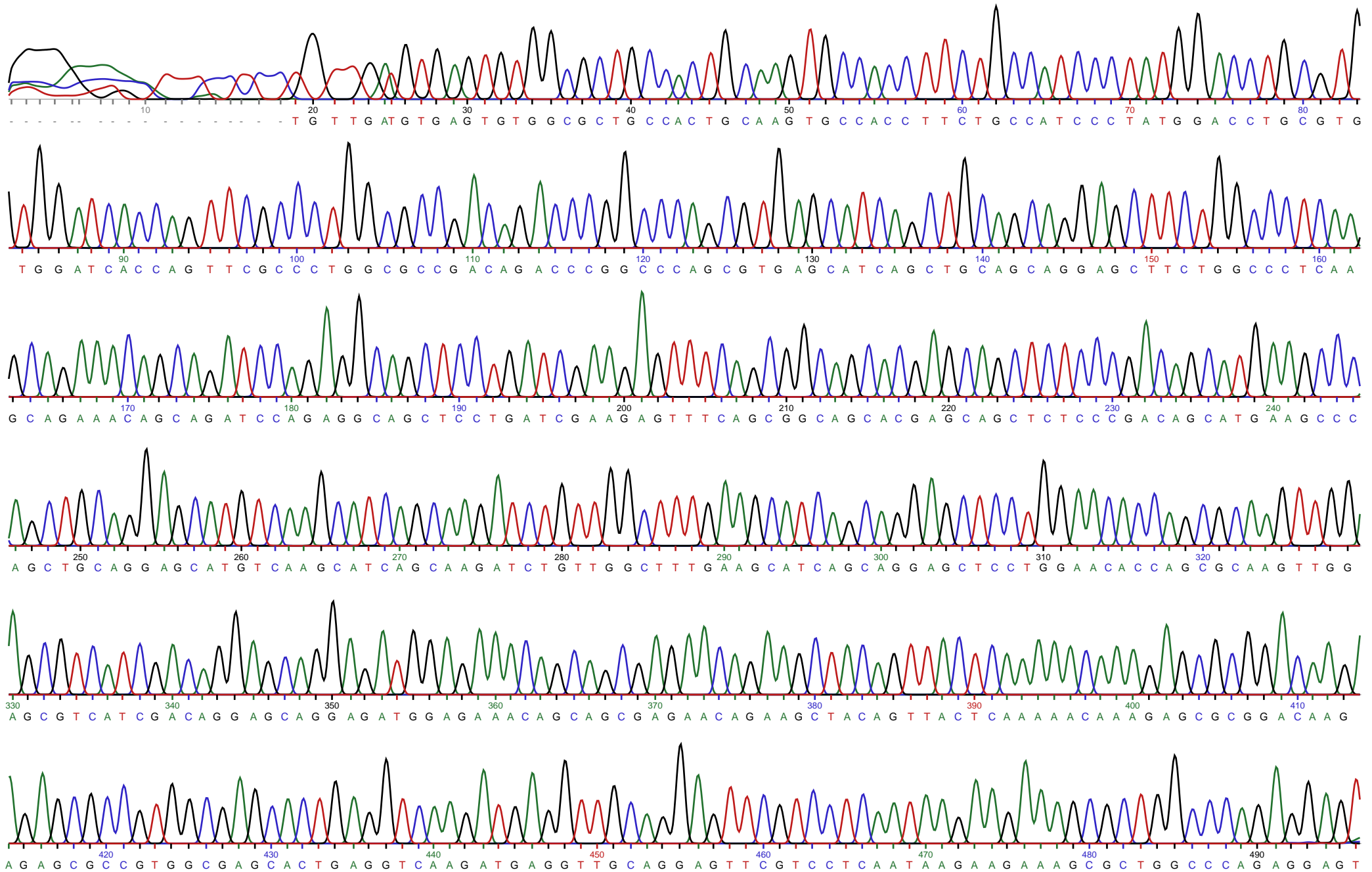

Samples: 19399  
Bases: 509  
Average spacing: 39

Page: 2 / 4  
6/30/2018

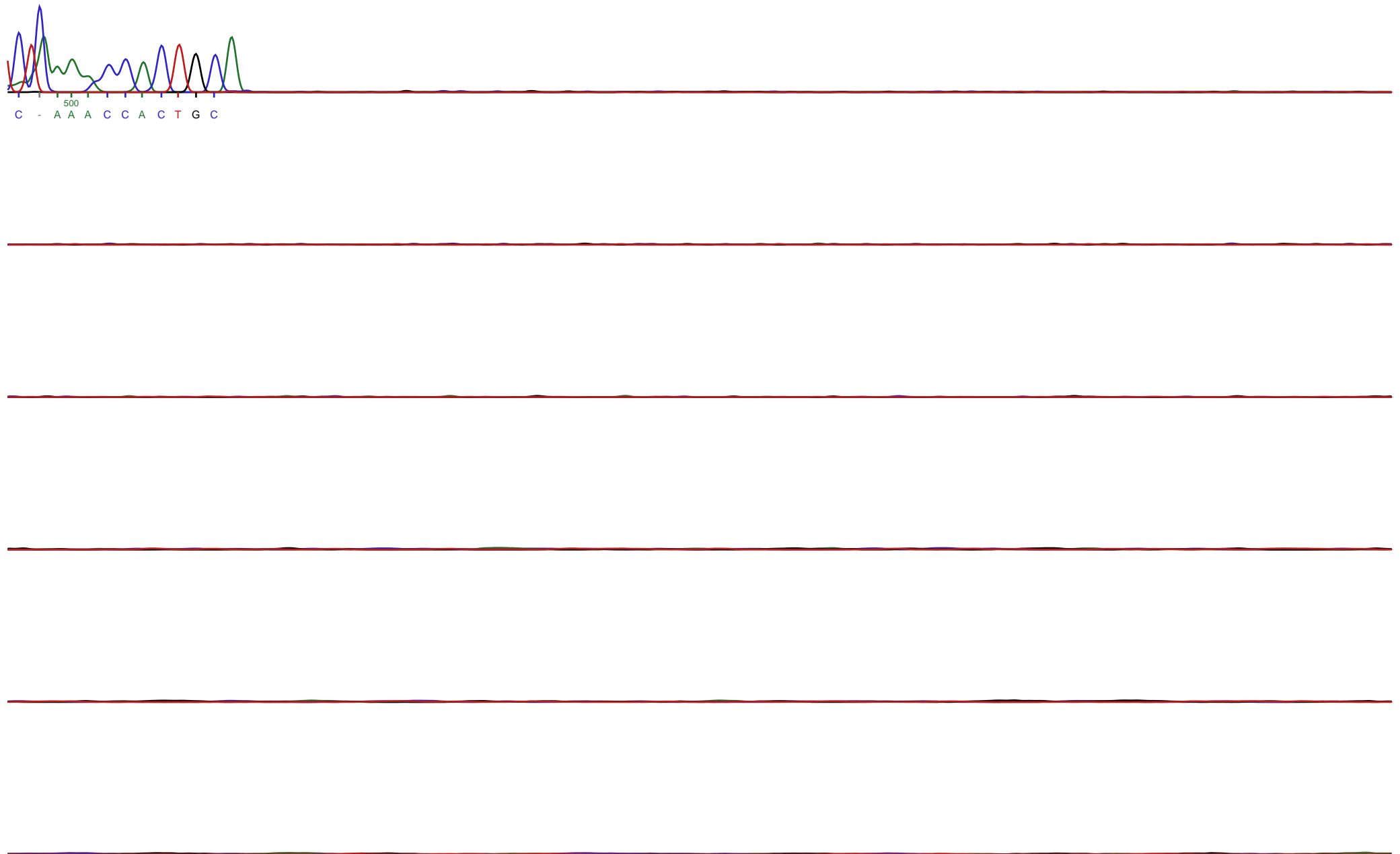

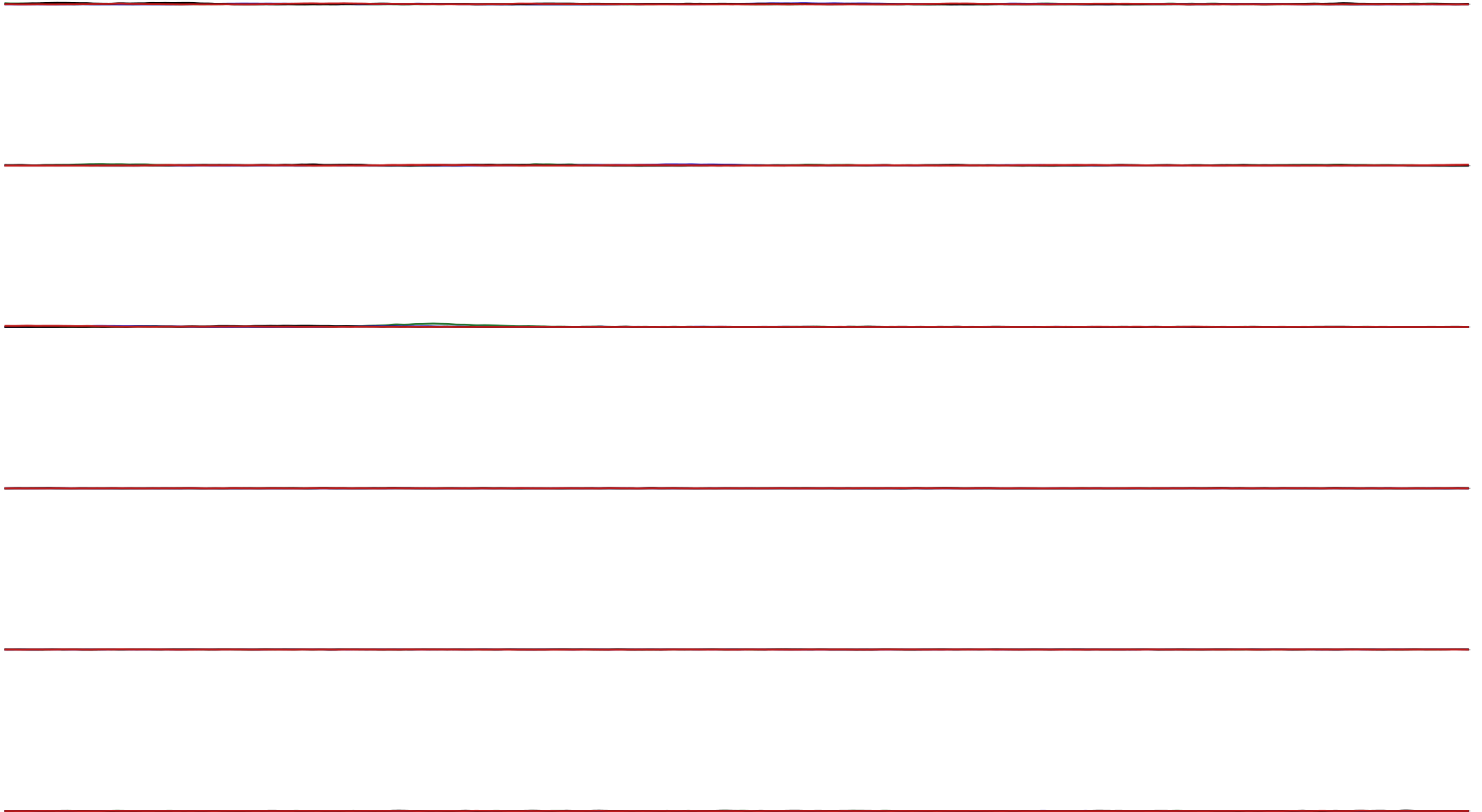

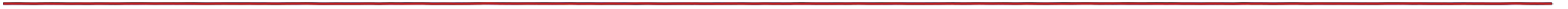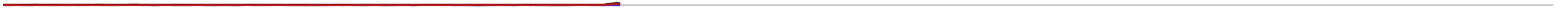

Supplement: Data S2 — Sequencing files of wild-type and mutant sibling cDNA sequenced from adult fin amputations. [file peerj-07-6167-s002.zip › WT3F_PREMIX_AJQV05_1.pdf]

Page: 1 / 3  
6/30/2018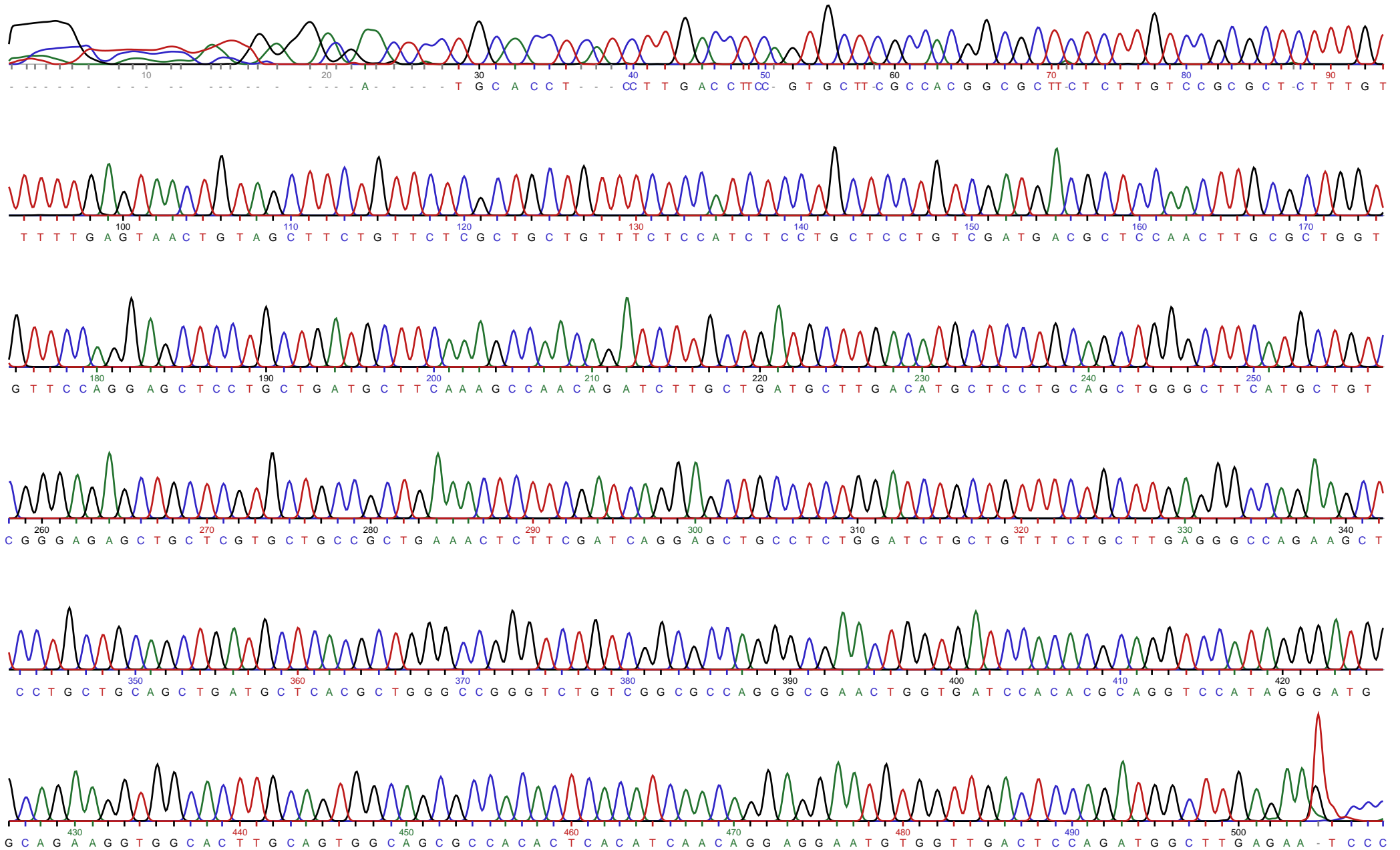

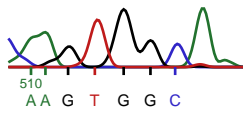

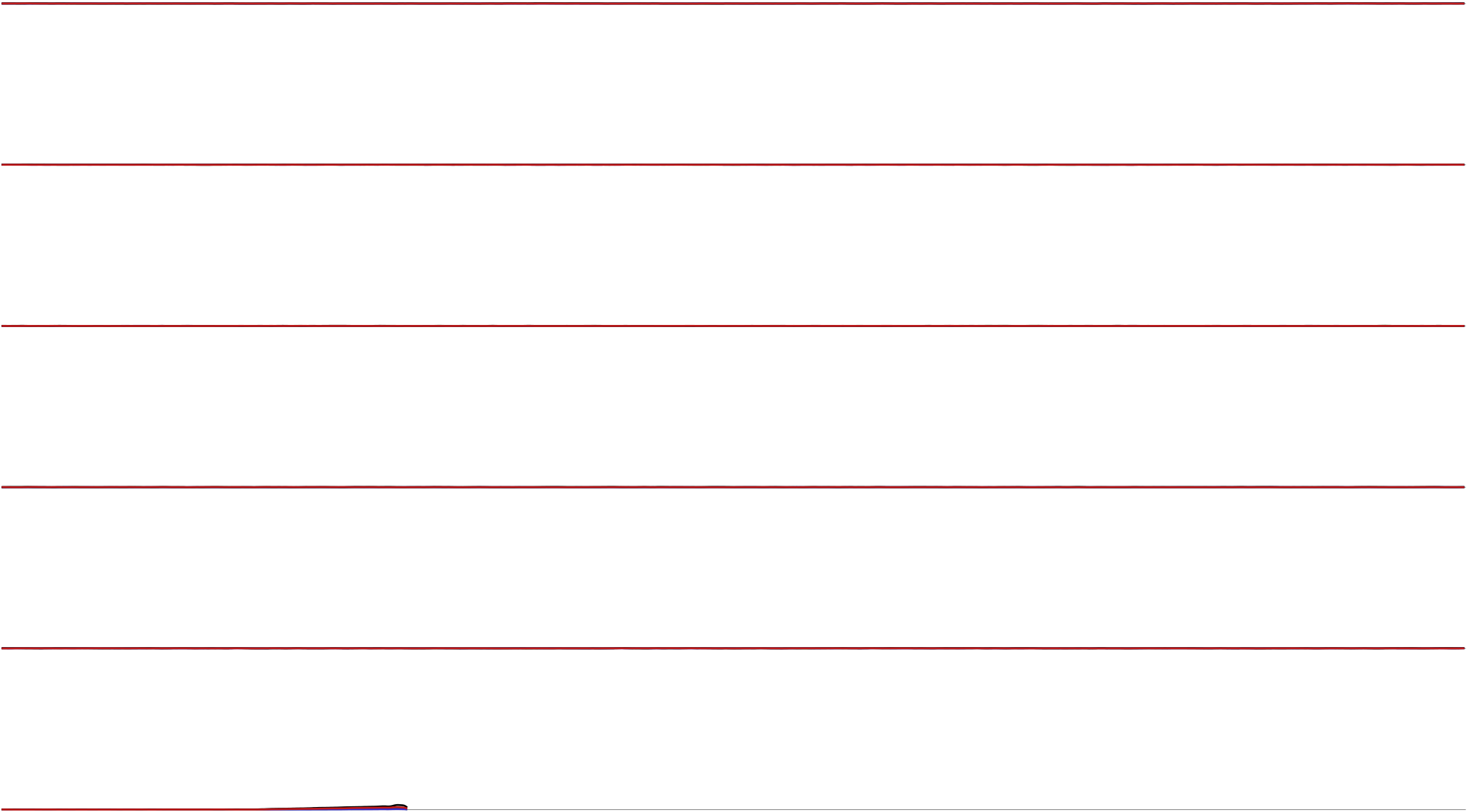

Supplement: Data S2 — Sequencing files of wild-type and mutant sibling cDNA sequenced from adult fin amputations. [file peerj-07-6167-s002.zip › WT6R_PREMIX_AJQV06_2.pdf]

Page: 1 / 3  
6/30/2018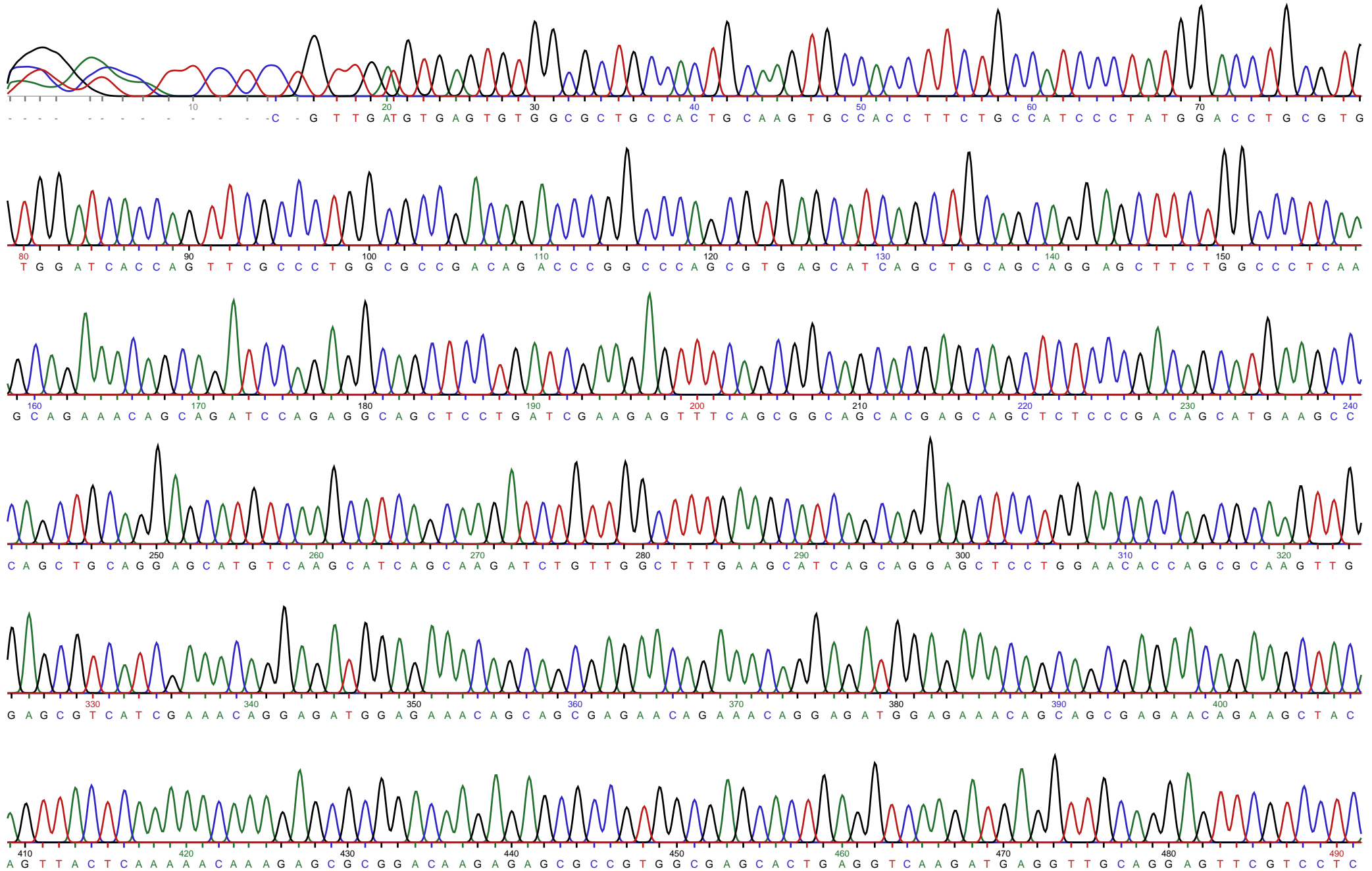

Samples: 17618  
Bases: 535  
Average spacing: 33

Page: 2 / 3  
6/30/2018

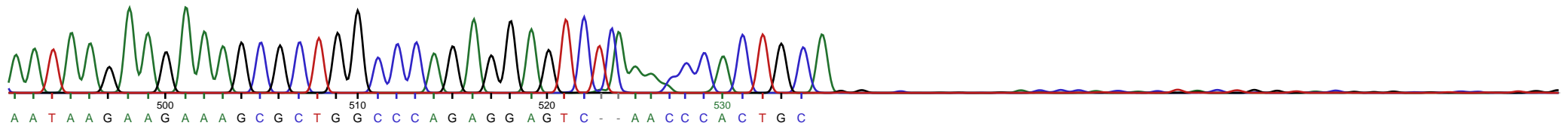

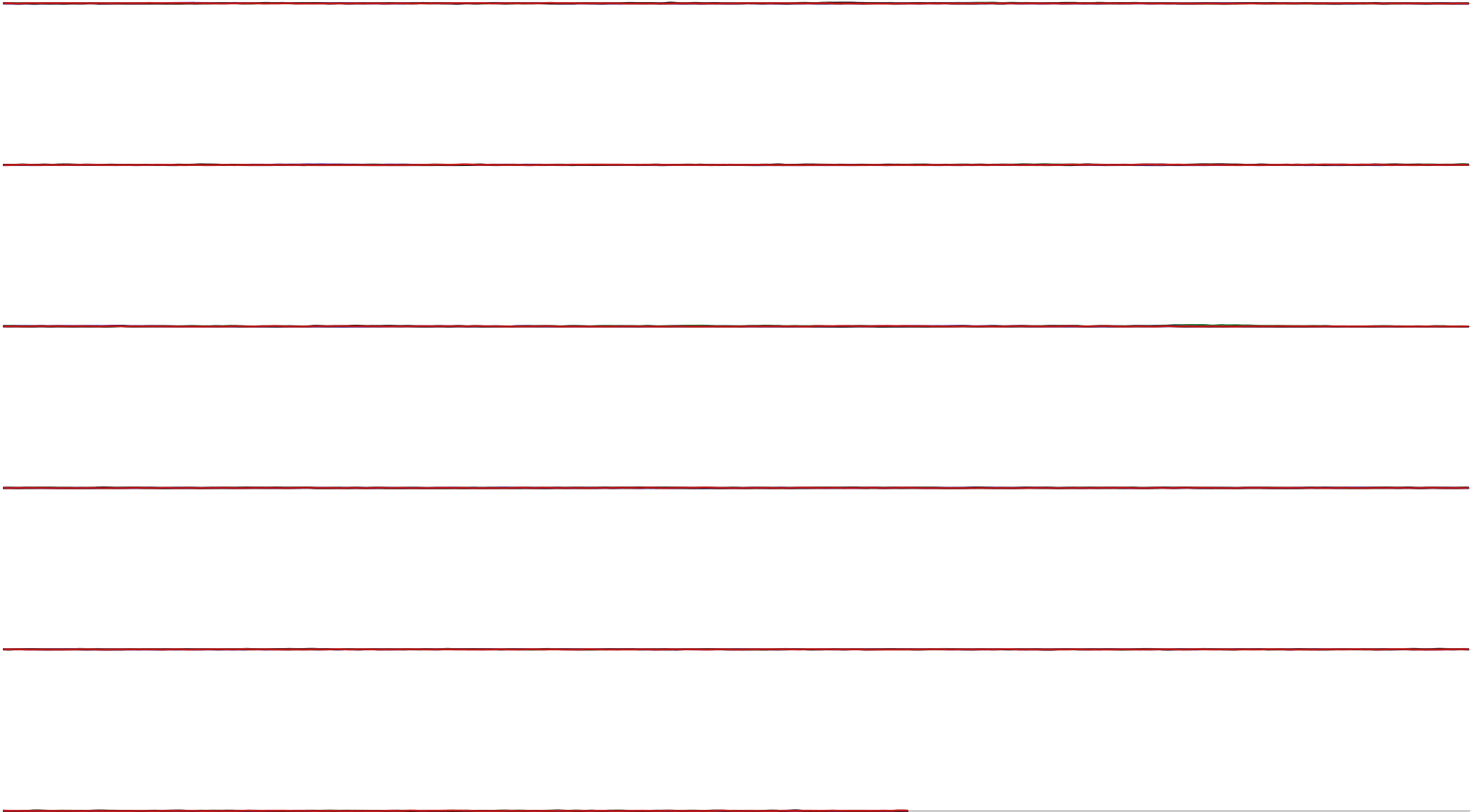

Supplement: Data S2 — Sequencing files of wild-type and mutant sibling cDNA sequenced from adult fin amputations. [file peerj-07-6167-s002.zip › mut3FL_PREMIX_AJQV09_5.pdf]

Page: 1 / 3  
6/30/2018

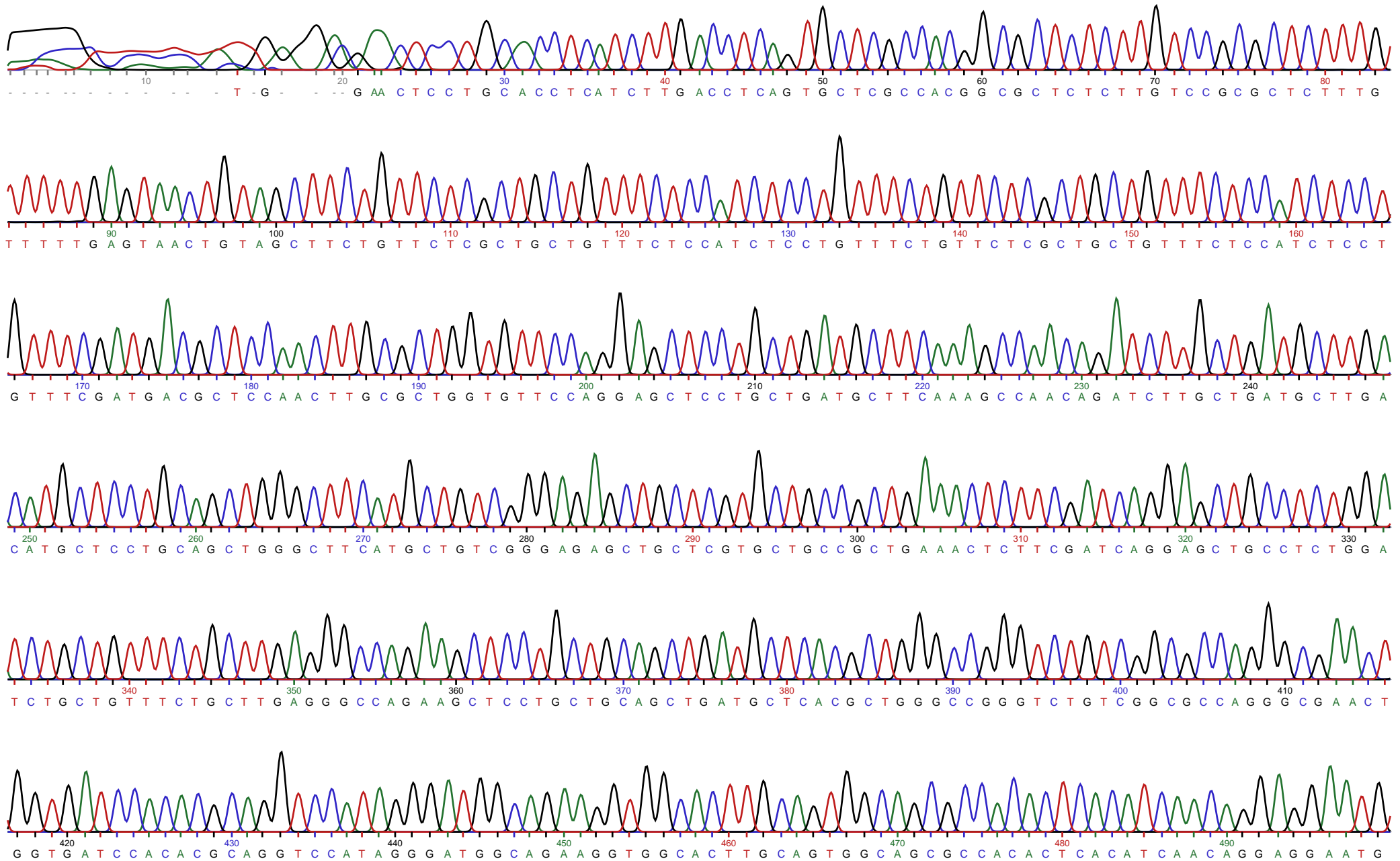

Samples: 15385  
Bases: 537  
Average spacing: 29

Page: 2 / 3  
6/30/2018

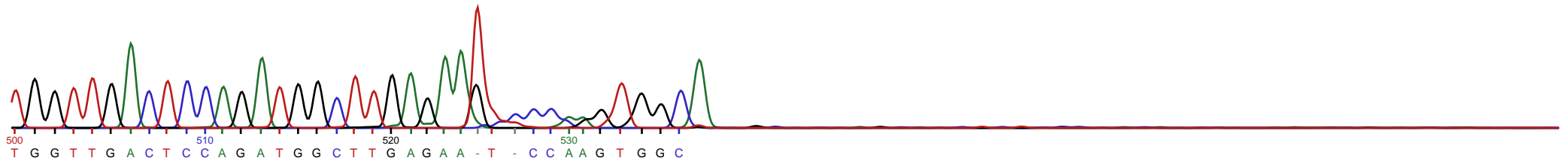

---

---

---

---

Supplement: Data S2 — Sequencing files of wild-type and mutant sibling cDNA sequenced from adult fin amputations. [file peerj-07-6167-s002.zip › mut6RL_PREMIX_AJQV10_6.pdf]
