# Supplementary material for: hdac4 mediates perichondral ossification and pharyngeal skeleton development in the zebrafish
Source: PeerJ. 2019 Jan 8;7:e6167. doi: 10.7717/peerj.6167 (PMC6329341; doi:10.7717/peerj.6167)

Samples: 15374
Bases: 532
Average spacing: 29

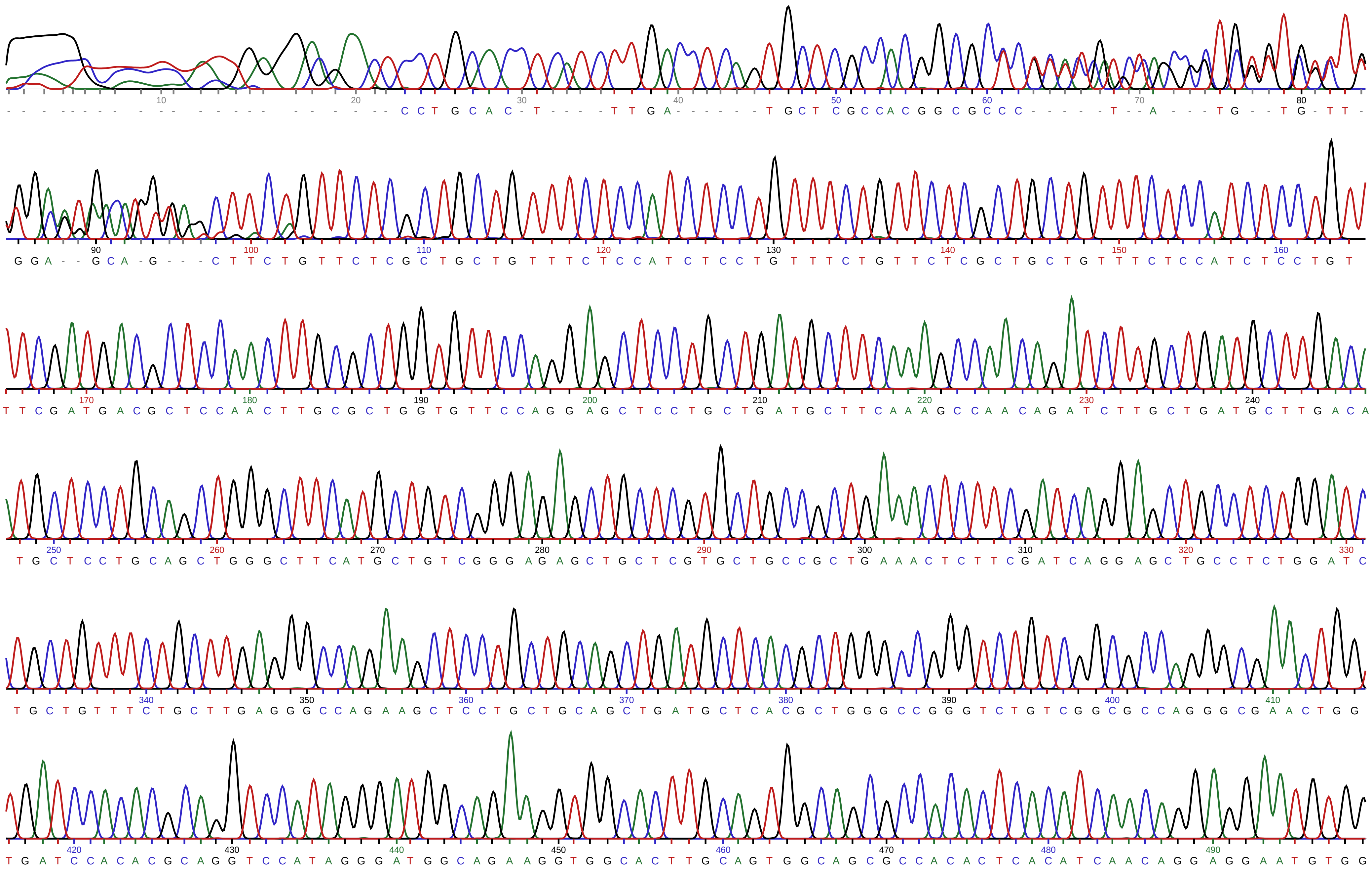

Samples: 15374
Bases: 532
Average spacing: 29

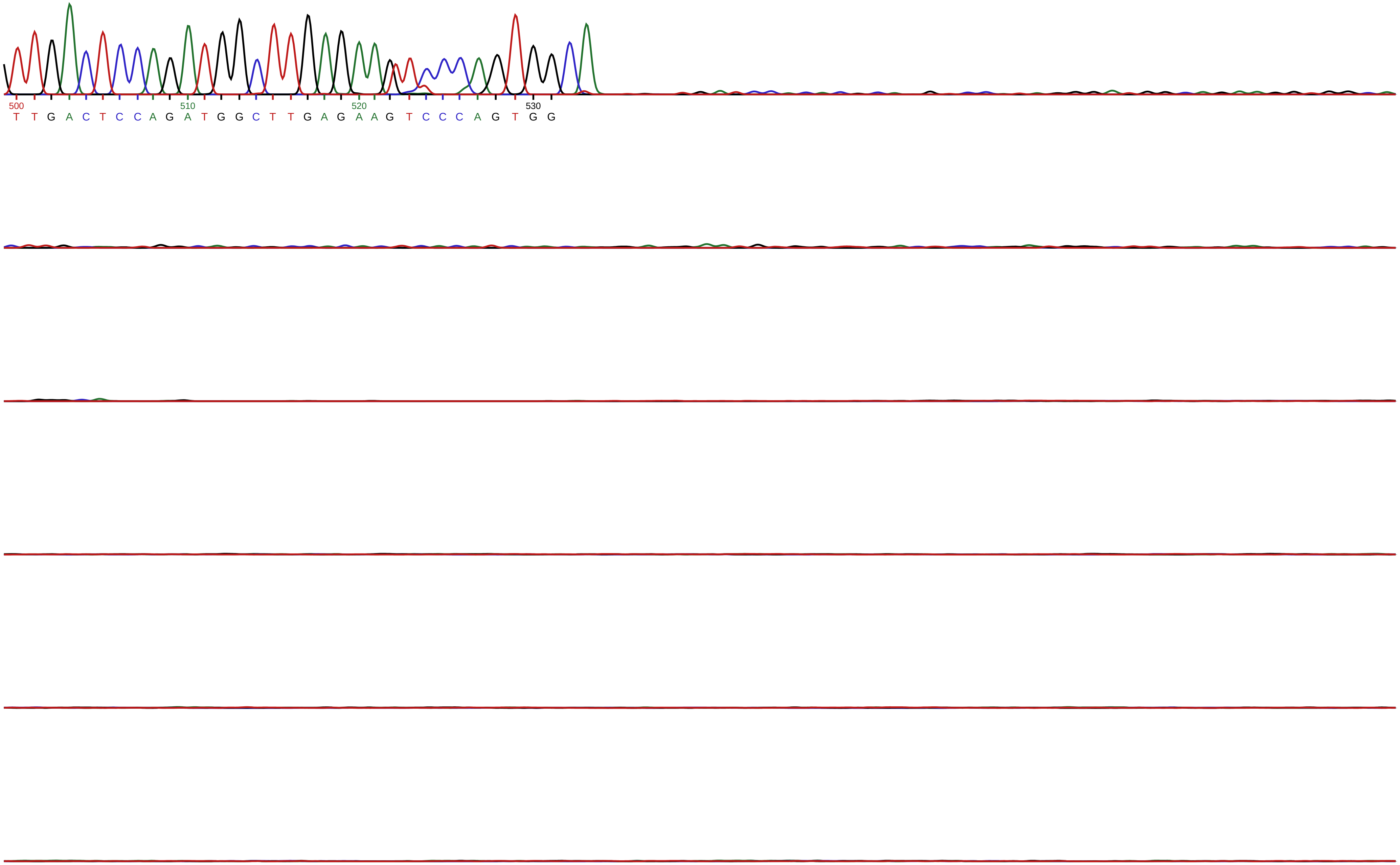

Samples: 15374
Bases: 532
Average spacing: 29

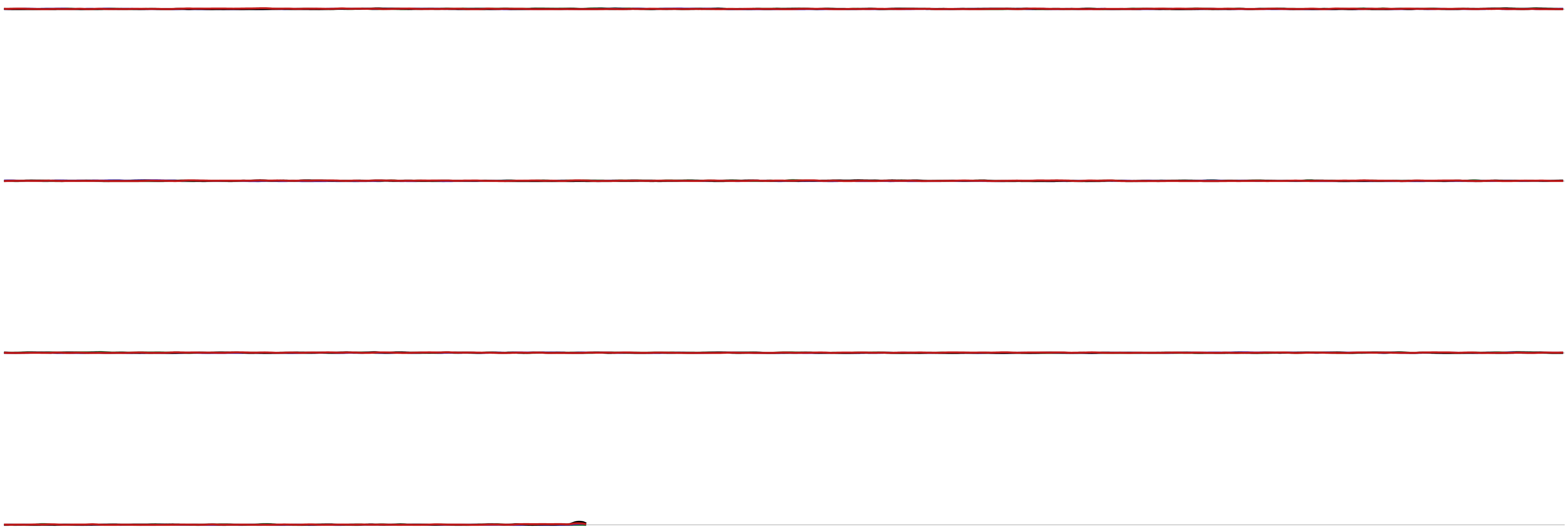

Supplement: Data S2 — Sequencing files of wild-type and mutant sibling cDNA sequenced from adult fin amputations. [file peerj-07-6167-s002.zip › mut6RH_PREMIX_AJQV07_3.pdf]
