# Supplementary material for: hdac4 mediates perichondral ossification and pharyngeal skeleton development in the zebrafish
Source: PeerJ. 2019 Jan 8;7:e6167. doi: 10.7717/peerj.6167 (PMC6329341; doi:10.7717/peerj.6167)

Samples: 13750
Bases: 657
Average spacing: 21

6/30/2018

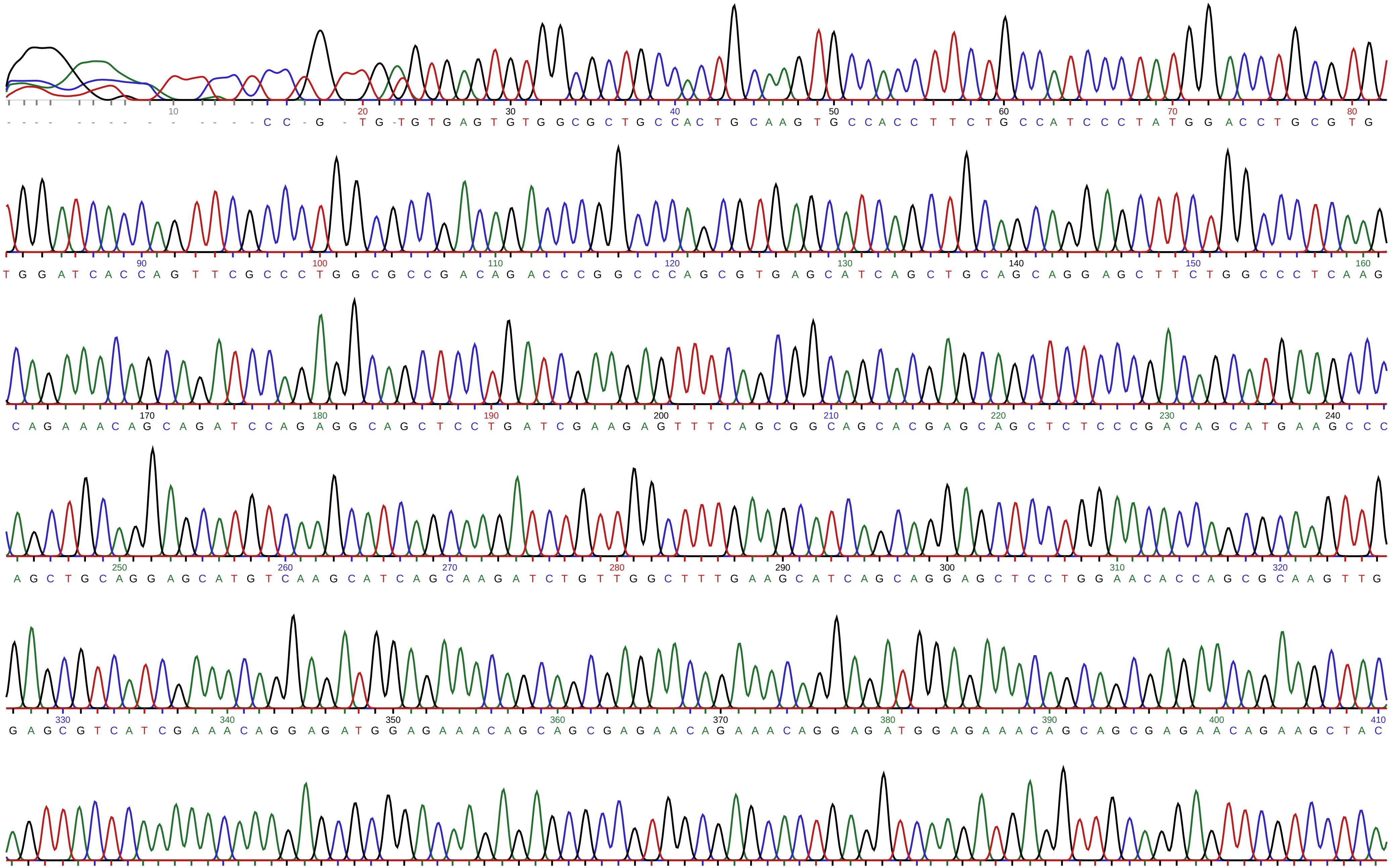

10 20 30 40 50 60 70 80
- - - - - - - - - - - - - - C C - G - T G -T G T G A G T G T G G C G C T G C C A C T G C A A G T G C C A C C T T C T G C C A T C C C T A T G G A C C T G C G T G

90 100 110 120 130 140 150 160
T G G A T C A C C A G T T C G C C C T G G C G C C G A C A G A C C C G G C C C A G C G T G A G C A T C A G C T G C A G C A G G A G C T T C T G G C C C T C A A G

170 180 190 200 210 220 230 240
C A G A A A C A G C A G A T C C A G A G G C A G C T C C T G A T C G A A G A G T T T C A G C G G C A G C A C G A G C A G C T C T C C C G A C A G C A T G A A G C C C

250 260 270 280 290 300 310 320
A G C T G C A G G A G C A T G T C A A G C A T C A G C A A G A T C T G T T G G C T T T G A A G C A T C A G C A G G A G C T C C T G G A A C A C C A G C G C A A G T T G

330 340 350 360 370 380 390 400 410
G A G C G T C A T C G A A A C A G G A G A T G G A G A A A C A G C A G C G A G A A C A G A A A C A G G A G A T G G A G A A A C A G C A G C G A G A A C A G A A G C T A C

420 430 440 450 460 470 480 490
A G T T A C T C A A A A A C A A A G A G C G C G G A C A A G A G A G C G C C G T G G C G A G C A C T G A G G T C A A G A T G A G G T T G C A G G A G T T C G T C C T C A

Samples: 13750
Bases: 657
Average spacing: 21

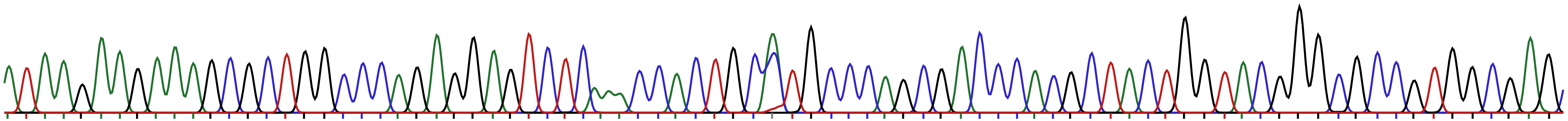

500 510 520 530 540 550 560 570

A T A A G A A G A A A G C G C T G G C C C A G A G G A G T C T C A A C C A C T G C - T G C C C A G C G A C C C A C G C T A C T G G T A C G G G C G C C G T G G C G A G

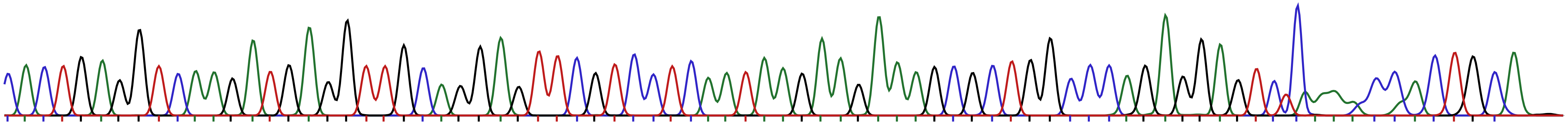

580 590 600 610 620 630 640 650

C A C T G A G G T C A A G A T G A G G T T G C A G G A G T T C G T C C T C A A T A A G A A G A A A G C G C T G G C C C A G A G G A G T C C A A A C C A C T G C

Samples: 13750
Bases: 657
Average spacing: 21

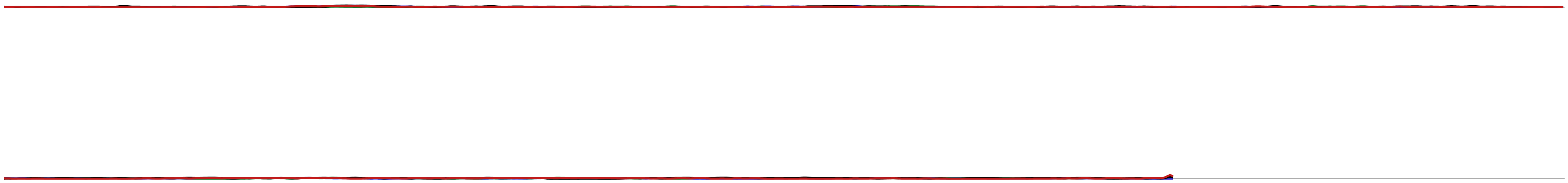

Supplement: Data S2 — Sequencing files of wild-type and mutant sibling cDNA sequenced from adult fin amputations. [file peerj-07-6167-s002.zip › mut3FH_PREMIX_AJQV08_4.pdf]
